# Can hydrolyzed silk fibroin derived from *Bombyx hatayensis* and ozonized gel modulate inflammatory and angiogenic responses in a corneal alkali burn model?

**DOI:** 10.55730/1300-0144.6372

**Published:** 2026-04-28

**Authors:** Şule BARMAN KAKİL, Hamdullah Suphi BAYRAKTAR, Okan SOYLU, İshak GÖKÇEK, Meryem İlkay KARAGÜL, Zehra DEMİRAY ASOĞLU

**Affiliations:** 1 Department of Ophthalmology, Tayfur Ata Sökmen Faculty of Medicine, Hatay Mustafa Kemal University, Hatay, Turkiye; 2 Experimental Research Application and Research Center, Hatay Mustafa Kemal University, Hatay, Turkiye; 3 Department of Physiology, Faculty of Veterinary Medicine, Hatay Mustafa Kemal University, Hatay, Turkiye; 4 Department of Histology and Embryology, Tayfur Ata Sökmen Faculty of Medicine, Hatay Mustafa Kemal University, Hatay, Turkiye

**Keywords:** Chemical corneal injury, *Bombyx hatayensis*, VEGF, neovascularization, biomaterials

## Abstract

**Background/aim:**

The therapeutic effects of hydrolyzed silk fibroin derived from *Bombyx hatayensis* and a commercially available ozonized gel, administered individually and in combination, were evaluated in a rat model of corneal alkali burn.

**Materials and methods:**

A standardized corneal alkali burn was induced in the right eyes of Wistar albino rats using 1 N sodium hydroxide (NaOH). Animals were randomly assigned to six groups: negative control, positive control (PC), hydrolyzed silk fibroin (SF), ozonized gel (O), combined treatment (SF + O), and antibiotic treatment. Topical treatments were administered for 21 consecutive days. Clinical evaluation included assessments of corneal opacity, neovascularization, and epithelial healing. On day 21, corneal tissues were harvested for biochemical analysis of TGF-β, VEGF, and TNF-α levels, as well as for histopathological assessment.

**Results:**

Significantly elevated levels of TGF-β, VEGF, and TNF-α were observed in untreated alkali-burned corneas compared with controls (p < 0.001). All treatment groups demonstrated significant reductions in these mediators compared with the PC group (p < 0.001). The SF and O groups showed comparable effects, whereas the SF + O group exhibited the lowest mean VEGF and TNF-α levels among the treatment groups, with no statistically significant difference compared with the antibiotic group (p > 0.05). Histopathological findings were consistent with the biochemical results, demonstrating improved epithelial integrity, reduced inflammatory cell infiltration, and more organized stromal architecture, particularly in the SF + O group.

**Conclusion:**

Hydrolyzed silk fibroin derived from *Bombyx hatayensis*, particularly when combined with ozonized gel, effectively attenuated inflammatory and angiogenic responses following corneal alkali injury. Outcomes comparable to those of conventional topical management were achieved with the combined therapy, with no statistically significant differences in biochemical and structural parameters, supporting its potential as a biocompatible, cell-free adjunctive strategy for severe chemical corneal burns.

## 1. Introduction

Corneal alkali burn is one of the most severe forms of ocular surface injury, characterized by intense inflammation and long-term structural complications. Among these complications, postinjury corneal neovascularization represents a major clinical challenge, as progressive vascular invasion compromises corneal transparency and visual function. Although corneal transplantation remains the definitive treatment for advanced cases, pathological blood and lymphatic vessel growth significantly increases the risk of immune-mediated graft rejection, thereby limiting long-term graft survival [1,2].

Accumulating experimental and clinical evidence highlights the central role of inflammatory mediators in the development of corneal neovascularization following alkali injury. Vascular endothelial growth factor (VEGF) is widely recognized as a key effector of angiogenesis in the injured cornea, whereas cytokines such as interleukin-1β (IL-1β) amplify inflammatory signaling and further enhance VEGF expression [3,4].

Experimental corneal alkali burn models have therefore been widely employed to investigate novel therapeutic strategies, as they reliably reproduce epithelial loss, stromal inflammation, neovascularization, and fibrotic remodeling. Despite advances in topical pharmacotherapy, conventional eye drop formulations are often limited by short ocular surface residence time and insufficient tissue penetration, resulting in suboptimal efficacy in severe burns. Consequently, increasing attention has been directed toward biomaterial-based approaches designed to stabilize the ocular surface microenvironment, prolong therapeutic exposure, and modulate postinjury inflammatory cascades. However, most previously investigated platforms rely on synthetic polymers or drug-loaded systems, and data on naturally derived, biocompatible alternatives—particularly those derived from region-specific biomaterials—remain limited [5].

Beyond angiogenic signaling, corneal alkali injury induces the rapid recruitment of inflammatory cells, including macrophages, which play a pivotal role in stromal damage and fibrotic remodeling. Sustained macrophage-driven cytokine release promotes extracellular matrix disorganization and persistent corneal opacity. Modulation of these inflammation-associated pathways during the subacute phase of corneal epithelial healing has therefore emerged as a critical therapeutic target for preserving corneal clarity [6].

Silk fibroin, a naturally derived protein obtained after the removal of sericin from silk cocoons, has gained considerable interest in regenerative medicine owing to its mechanical strength, biocompatibility, biodegradability, and low immunogenicity. Its hierarchical molecular structure enables its processing into films, sponges, nanofibers, and hydrogels with tunable physicochemical properties. In hydrogel form, silk fibroin mimics the extracellular matrix by providing a hydrated three-dimensional environment that supports cell adhesion and tissue repair while maintaining optical transparency and oxygen permeability, features that are particularly relevant for ocular surface applications [7].

In parallel, ozone therapy has emerged as a potential adjunctive modality for ocular surface injuries due to its antimicrobial, antiinflammatory, and antioxidant effects. Experimental data suggest that topical ozone can improve tissue oxygenation, attenuate oxidative stress, and reduce epithelial and stromal damage in chemical burn models. Nevertheless, most existing evidence derives from acidic injury models, and the therapeutic role of ozone in alkali-induced corneal damage, particularly in combination with regenerative biomaterials, remains insufficiently explored [8].

In this context, the therapeutic effects of hydrolyzed silk fibroin derived from *Bombyx hatayensis*, an endemic silkworm species native to Türkiye, and a commercially available ozonized gel, administered individually and in combination, were evaluated in a rat model of corneal alkali burn. To our knowledge, the ocular surface effects of hydrolyzed silk fibroin obtained from *Bombyx hatayensis* have not previously been investigated in this injury model. Treatment efficacy was assessed using clinically relevant parameters, including epithelial defect closure, corneal opacity, and neovascularization, alongside quantitative evaluation of inflammatory and angiogenic mediators. By comparing single and combined therapies, this study sought to explore potential additive effects and to provide experimental evidence supporting the use of naturally derived, biocompatible biomaterials as adjunctive strategies for severe chemical corneal burns.

## 2. Materials and methods

All experimental procedures involving animals were conducted in accordance with the ARVO Statement for the Use of Animals in Ophthalmic and Vision Research. The study protocol was approved by the Animal Experiments Local Ethics Committee of Hatay Mustafa Kemal University (decision no: 2025/05-09; meeting date: 24 June 2025; document no: E-40595970-050.04-523910; dated 02 July 2025). All efforts were made to minimize animal suffering and to reduce the number of animals used, in accordance with the 3R principles.

### 2.1. Animals and study design

Adult male Wistar albino rats (250–300 g) were housed under standardized laboratory conditions (12 h light/dark cycle with ad libitum access to food and water). Animals were randomly allocated into six groups using a computer-generated randomization sequence performed by an investigator not involved in outcome assessment. The negative control (NC) group consisted of three rats and received no corneal injury. The positive control (PC) group included seven rats subjected to alkali burn without treatment. The silk fibroin (SF) group (n = 7) underwent alkali burn followed by treatment with topical hydrolyzed silk fibroin. The ozonized gel (O) group (n = 7) underwent alkali burn followed by treatment with topical ozonized gel. The combination (SF + O) group (n = 7) received both hydrolyzed silk fibroin and ozonized gel following alkali injury. The antibiotic treatment (ANT) group (n = 7) underwent alkali burn followed by treatment with topical antibiotic and artificial tear therapy. The smaller NC group size was selected in accordance with ethical reduction principles, as this group was expected to demonstrate minimal biological variability. Clinical and histopathological evaluations were performed by investigators masked to treatment allocation.

### 2.2. Anesthesia procedure

General anesthesia was induced via intraperitoneal injection of ketamine hydrochloride (70–80 mg/kg) and xylazine hydrochloride (8–10 mg/kg). The administered doses were adjusted according to body weight for each animal. Adequate depth of anesthesia was confirmed before all experimental procedures based on physiological responses.

### 2.3. Experimental corneal alkali burn model

A standardized alkali burn was induced in the right eye of each rat. A 3-mm diameter Whatman no. 3 filter paper disc soaked with 25 μL of 1 N NaOH was applied centrally to the cornea for 30 s without external pressure. The filter paper was kept stationary during application to avoid peripheral tissue injury. Immediately after removal, the ocular surface was irrigated with 20 mL of sterile 0.9% saline over 60 s. Undesirable conjunctival adhesions were prevented through irrigation of the eye. Excess saline was gently wiped from the eye and surrounding tissues using a disposable soft wipe. Animals were monitored daily for the first 3 days. Subcutaneous meloxicam (4–6 mg/kg) was administered when signs of discomfort were observed [9].

### 2.4. Experimental groups and treatments

Following induction of corneal alkali burn, animals were allocated to six experimental groups. The negative control (NC) group received no corneal injury. The PC group underwent alkali burn without any subsequent treatment. The silk fibroin (SF) group received treatment with topical hydrolyzed silk fibroin (10 μL per application) following alkali injury, whereas the ozonized gel (O) group received treatment with topical ozonized gel (10 μL per application). In the combination (SF + O) group, animals received both hydrolyzed silk fibroin and ozonized gel administered sequentially. The antibiotic treatment (ANT) group received treatment with topical 0.3% tobramycin and sodium hyaluronate artificial tear drops as conventional supportive therapy aimed at preventing secondary bacterial infection following alkali injury. All topical treatments were administered three times daily for 21 consecutive days.

### 2.5. Preparation of hydrolyzed silk fibroin

**Figure 1:**
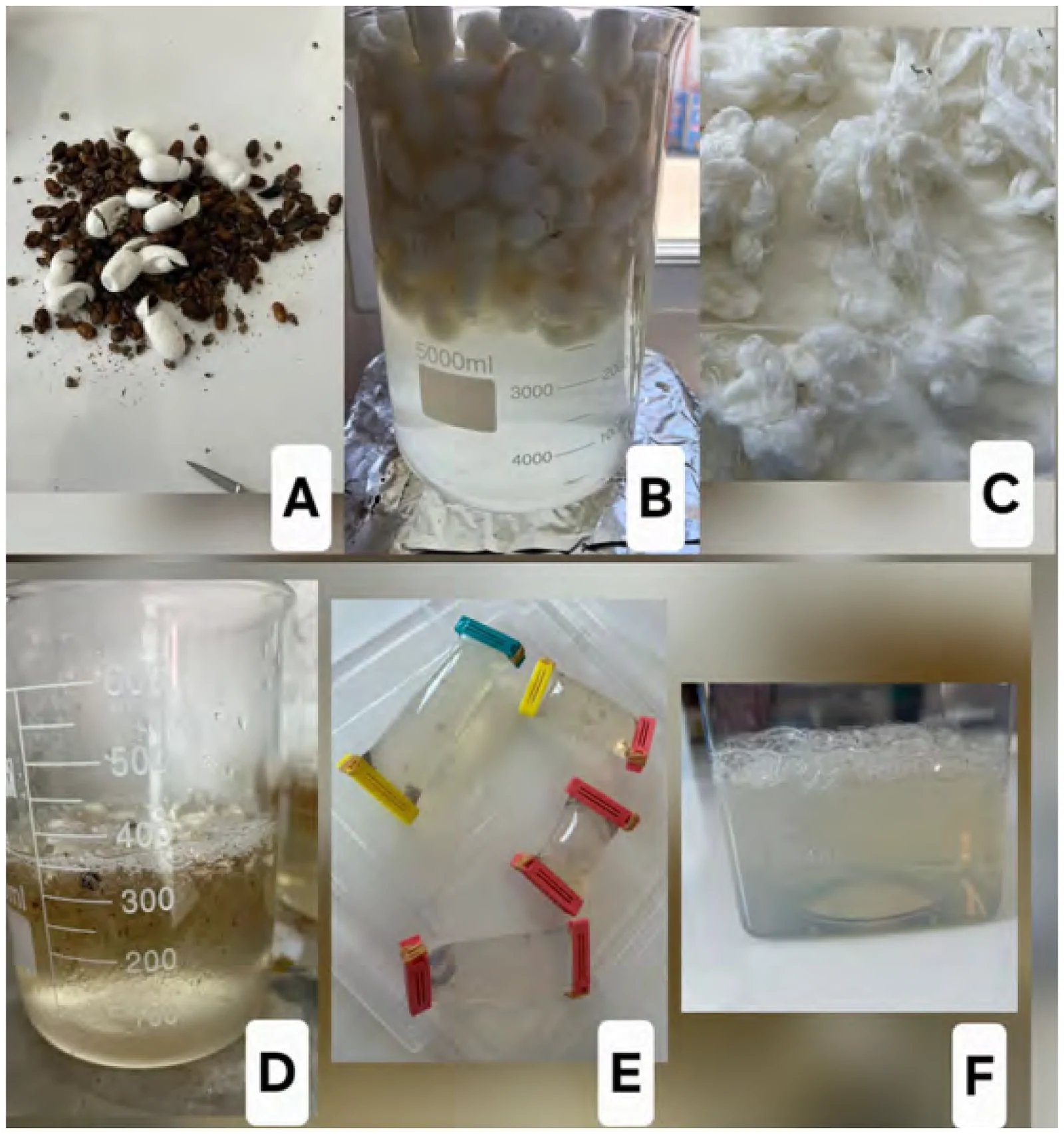
Preparation of hydrolyzed silk fibroin derived from *Bombyx hatayensis* cocoons. (A) Raw silk cocoons prior to processing. (B) Degumming process for the removal of sericin. (C) Dried silk fibroin fibers following degumming. (D) Dissolution of silk fibroin fibers in a lithium bromide solution. (E) Purification by dialysis using a 10 kDa molecular weight cut-off membrane. (F) Final sterile hydrolyzed silk fibroin solution prepared for topical application (70 mg/mL).

Raw silk cocoons obtained from *Bombyx hatayensis*, an endemic silkworm species native to Türkiye, were used as the source material (Figure 1A). The cocoons were first subjected to a degumming process to remove sericin (Figure 1B), followed by thorough washing with distilled water. After degumming, the fibroin fibers were air-dried at room temperature until complete removal of residual moisture (Figure 1C).

Subsequently, the degummed and dried silk fibroin was transferred into a lithium bromide (LiBr) solution to induce dissolution (Figure 1D). The fibroin–LiBr mixture was incubated in a 60 °C drying oven for 4 h, during which complete dissolution of the silk fibroin was achieved, resulting in a homogeneous solution.

Following dissolution, the silk fibroin solution was transferred into dialysis tubing with a 10 kDa molecular weight cutoff and dialyzed against distilled water for 4 days with regular water changes to remove residual LiBr and low-molecular-weight impurities (Figure 1E). At the end of the dialysis process, the purified silk fibroin solution was collected and transferred into sterile containers under aseptic conditions.

The final concentration of the hydrolyzed silk fibroin solution was adjusted to 70 mg/mL and stored until topical application (Figure 1F).

### 2.6. Ozonized gel

A commercially available ozonized gel (commercial product; details withheld for blinded peer review) was used as the ozone-based treatment in this study.

The product was supplied in liquid gel form and is based on ozonized compounds (ozonides), typically derived from ozonized oils, which are known to possess antimicrobial, antiinflammatory, and oxidative stress–modulating properties.

According to the manufacturer’s specifications, the formulation is intended for topical application and has been previously used in experimental and clinical contexts for its biological activity. Although precise quantitative information regarding the ozone concentration was not disclosed by the manufacturer, this limitation is acknowledged.

The ozonized gel was stored and handled in accordance with the manufacturer’s recommendations throughout the study period. For topical administration, a standardized volume was applied directly to the injured corneal surface three times daily under sterile conditions.

### 2.7. Treatment administration

For topical administration, 10 μL of hydrolyzed silk fibroin solution or ozonized gel was applied directly to the corneal surface using a sterile micropipette. In the combined treatment group (SF + O), hydrolyzed silk fibroin and ozonized gel were administered sequentially, with an interval of approximately 5 min between applications to minimize potential washout effects and ensure adequate ocular surface contact. All applications were performed under sterile conditions.

### 2.8. Clinical evaluation

Clinical evaluation was performed on days 1, 4, 7, and 21 following alkali injury. Corneal opacity, stromal edema, and neovascularization were assessed by slit-lamp biomicroscopy under standardized white-light illumination. Corneal opacity was graded using the 0–4 scoring system described by Yoeruek et al. [10]. In this scale, grade 0 indicates a completely clear cornea; grade 1 indicates slight haze with the iris and pupil clearly visible; grade 2 indicates moderate opacity with the iris and pupil visible; grade 3 indicates marked opacity with difficulty visualizing the pupil; and grade 4 indicates complete corneal opacity with no visible pupil. Corneal neovascularization was evaluated by slit-lamp biomicroscopy under standardized white-light illumination and graded semiquantitatively according to the centripetal extent of newly formed vessels extending from the limbus toward the central cornea: grade 0, no visible neovascularization; grade 1, vessels extending <25% of the corneal radius; grade 2, vessels extending 25%–50% of the corneal radius; grade 3, vessels extending 50%–75% of the corneal radius; and grade 4, vessels extending >75% of the corneal radius or reaching the central cornea. All clinical scoring was performed independently by two masked observers, and a consensus score was recorded in cases of disagreement. For epithelial defect assessment, topical fluorescein sodium was instilled, and the cornea was examined under cobalt-blue illumination. In cases of disagreement, a consensus score was recorded. Representative digital images were obtained for documentation and comparative analysis.

### 2.9. Biochemical analysis

On day 21, corneas were excised, homogenized in phosphate-buffered saline, and centrifuged. Supernatants were collected for enzyme-linked immunosorbent assay (ELISA) analysis. TGF-β, VEGF, and TNF-α levels were quantified using commercially available ELISA kits according to the manufacturer’s instructions. Cytokine concentrations were normalized to total protein content and expressed in ng/mL.

### 2.10. Histopathological evaluation

Corneas were fixed in 10% formalin, paraffin embedded, sectioned (4 μm), and stained with hematoxylin and eosin (H&E). Slides were evaluated by a blinded histologist. Stromal edema, inflammatory cell infiltration, and neovascularization were assessed descriptively.

### 2.11. Euthanasia and tissue collection

On day 21, all animals were euthanized under deep anesthesia. The eyes were enucleated, and the corneas were carefully excised using microsurgical scissors. Corneal tissues were fixed in 10% neutral buffered formalin for further analysis. All samples were coded and evaluated in a blinded manner.

### 2.12. Statistical analysis

Statistical analyses were performed using SPSS (version 26; IBM Corp., Armonk, NY, USA). Data normality was assessed using the Shapiro–Wilk test. For comparisons among groups, one-way ANOVA with Tukey’s post hoc test was used for normally distributed data with homogeneity of variances; when the assumption of homogeneity of variances was not met, Welch ANOVA with the Games–Howell post hoc test was applied. Effect sizes (η^2^, eta-squared) were calculated to assess the magnitude of group differences. Where appropriate, 95% confidence intervals (CIs) were also reported to improve interpretability. Data are presented as mean ± standard deviation. A p < 0.05 was considered statistically significant.

## 3. Results

### 3.1. Corneal opacity

Corneal opacity and neovascularization scores are summarized in Table.

Significant intergroup differences were observed from day 4 onward for both parameters (overall p < 0.0001).

On day 1, all alkali-burn groups (PC, SF, O, SF + O, and ANT) demonstrated marked corneal haze with minimal neovascularization, with no statistically significant differences among groups (p = 0.74 and p = 0.81, respectively), reflecting the acute inflammatory response following chemical injury.

By day 4, both corneal opacity and neovascularization scores remained highest in the PC group, whereas the treatment groups exhibited partial improvement. The SF and O groups showed moderate reductions in both parameters compared with the PC group (p = 0.032–0.041). The SF + O and ANT groups demonstrated more pronounced improvement; however, no statistically significant difference was observed between these two groups (p = 0.58).

On day 7, intergroup differences became more evident (p < 0.0001). The PC group maintained high opacity and neovascularization scores, consistent with persistent stromal inflammation and a progressive angiogenic response. The SF and O groups exhibited moderate opacity (typically grade 2–3) and intermediate neovascularization. In contrast, the SF + O and ANT groups showed significantly lower scores compared with the PC group (p < 0.001) and lower mean values compared with the SF and O groups (p = 0.018), with no statistically significant difference between the SF + O and ANT groups (p = 0.62).

By day 21, corneal opacity and neovascularization remained markedly elevated in the PC group, consistent with stromal scarring and established vascular ingrowth. The SF and O groups demonstrated partial improvement, although residual pathological findings persisted. The SF + O and ANT groups exhibited the lowest scores among the injured groups, indicating improved corneal clarity and reduced vascular invasion. No statistically significant difference was observed between the SF + O and ANT groups (p = 0.67), whereas both groups showed significantly lower scores compared with the PC group (p < 0.001). As expected, the NC group maintained grade 0 opacity and absence of neovascularization throughout the study period.

Overall, combination therapy (SF + O) resulted in a more pronounced reduction in both corneal opacity and neovascularization compared with the single-treatment groups and outcomes similar to those of conventional supportive therapy, with no statistically significant difference observed between the SF + O and ANT groups by day 21.

**Figure 2:**
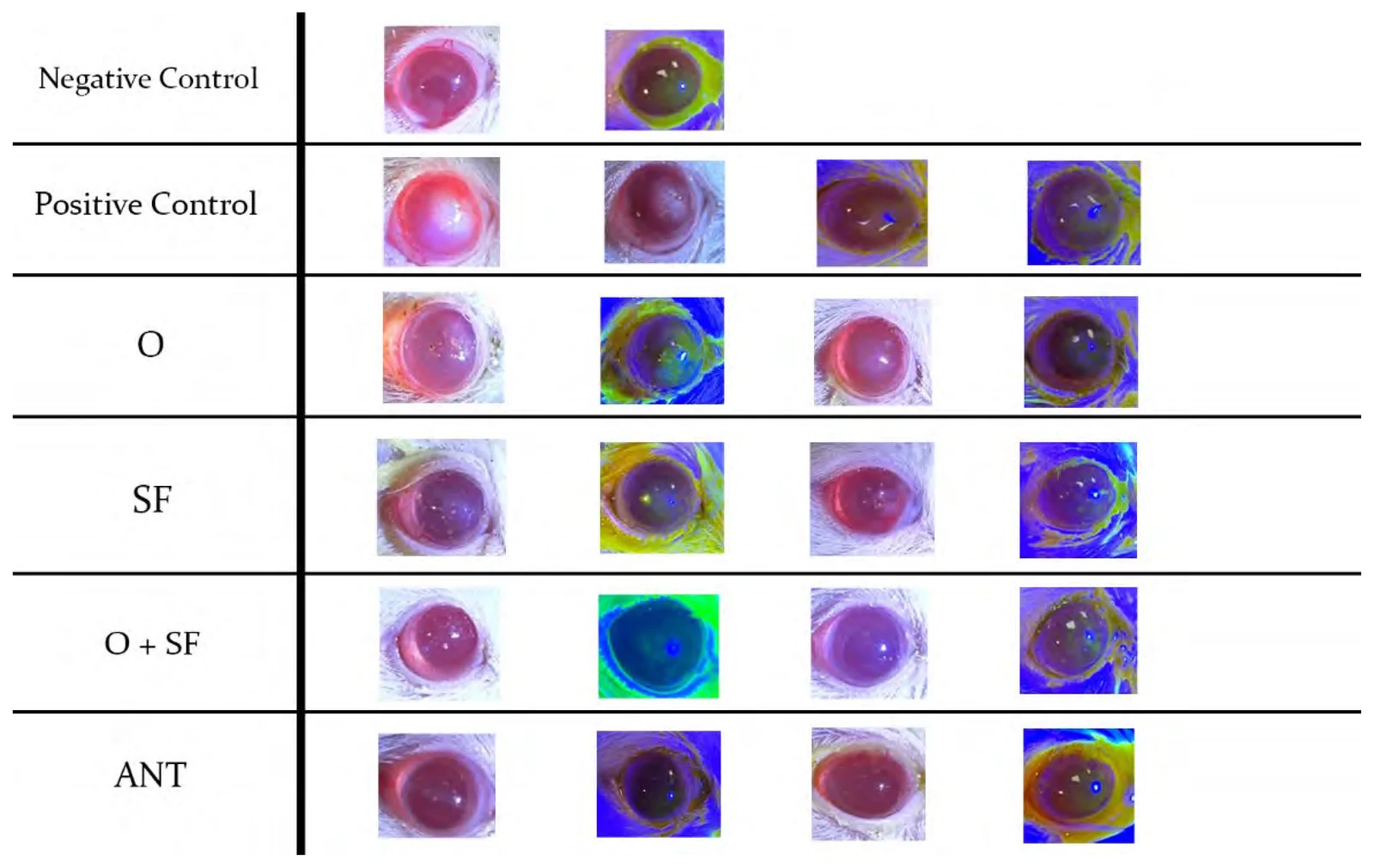
Representative clinical images of corneal healing following alkali injury. For each treatment group, images are presented in the following order: day 7 white-light (unstained), day 7 fluorescein staining under cobalt-blue illumination, day 21 white-light (unstained), and day 21 fluorescein staining. Fluorescein-positive areas indicate epithelial defects. The negative control (NC) group demonstrates normal corneal transparency and absence of epithelial defects. The positive control (PC) group demonstrates persistent corneal opacity, stromal haze, and marked epithelial defects, particularly evident at day 21. The silk fibroin (SF) and ozone (O) groups exhibit partial improvement with residual opacity and reduced but persistent epithelial staining. The combination (SF + O) group demonstrates greater corneal clarity and markedly reduced fluorescein staining over time. The antibiotic (ANT) group demonstrates comparable epithelial recovery and reduced opacity by day 21. The images are representative of each experimental group.

Representative macroscopic white-light and fluorescein-stained images obtained under cobalt-blue illumination on days 7 and 21 further demonstrate differences in corneal opacity and epithelial defect resolution among the groups (Figure 2).

### 3.2. Biochemical findings

**Figure 3:**
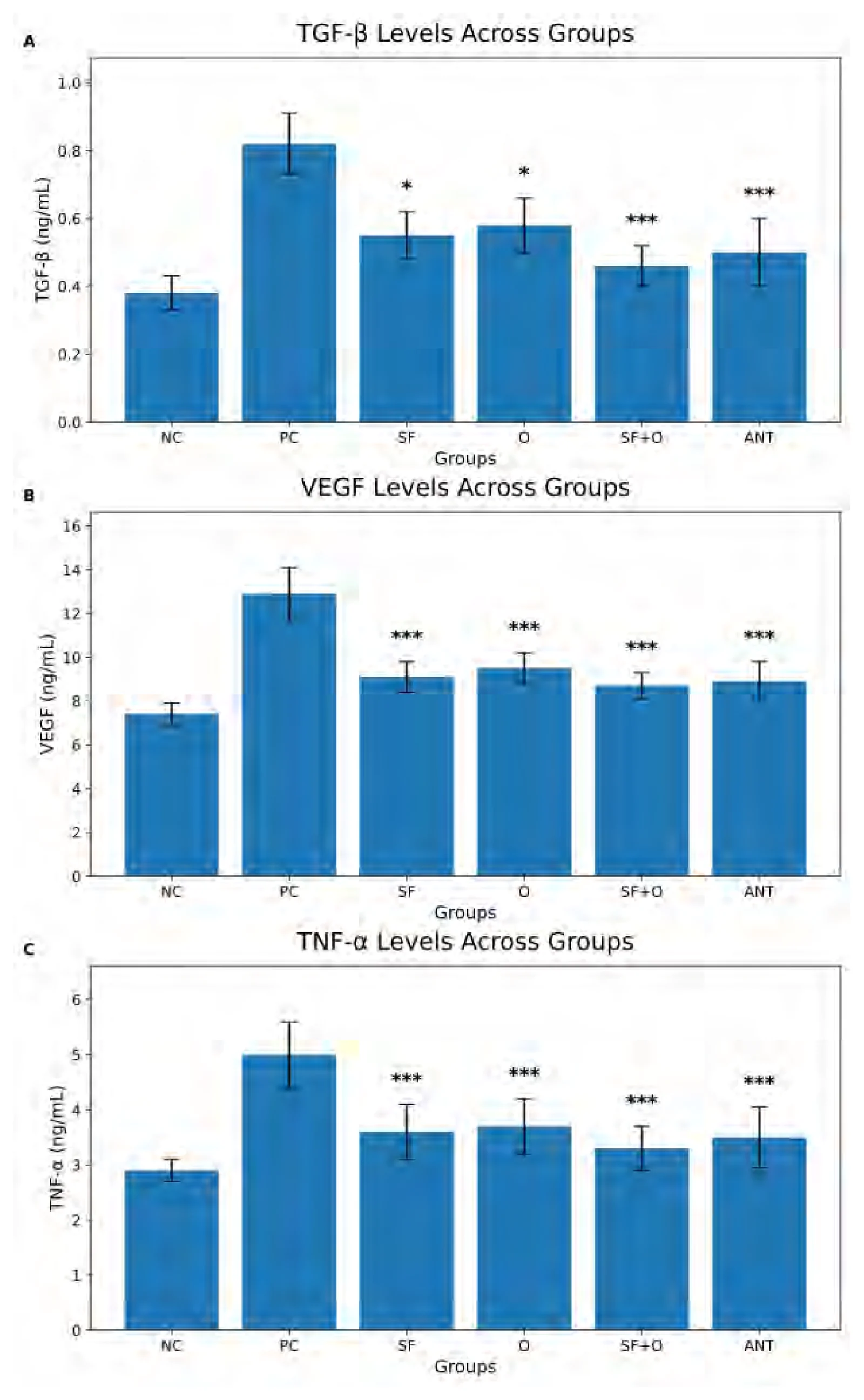
Comparison of corneal TGF-β, VEGF, and TNF-α levels among the experimental groups. *p < 0.05, **p < 0.01, ***p < 0.001 compared with the positive control (PC) group.

Quantitative analyses of inflammatory and angiogenic mediators are presented in Figure 3. A statistically significant difference in TGF-β levels was observed among the groups (one-way ANOVA; p < 0.001). The highest TGF-β level was detected in the positive control (PC) group (0.823 ± 0.091 ng/mL), whereas the lowest level was observed in the negative control (NC) group (0.382 ± 0.047 ng/mL). The SF (0.548 ± 0.072 ng/mL) and O (0.579 ± 0.081 ng/mL) groups showed comparable TGF-β levels, falling between the NC and PC groups. No statistically significant difference was found between the SF + O group (0.463 ± 0.058 ng/mL) and the antibiotic treatment group (ANT) (0.502 ± 0.096 ng/mL) (p > 0.05). Compared with the PC group, all treatment groups demonstrated a statistically significant reduction in TGF-β levels (p < 0.001).

VEGF levels also differed significantly among the groups (p < 0.001). The highest VEGF level was measured in the PC group (12.850 ± 1.200 ng/mL), whereas the lowest value was observed in the NC group (7.420 ± 0.480 ng/mL). Compared with the PC group, VEGF levels were significantly reduced in the SF (9.120 ± 0.650 ng/mL), O (9.450 ± 0.700 ng/mL), SF + O (8.680 ± 0.600 ng/mL), and ANT (8.920 ± 0.850 ng/mL) groups (p < 0.001). Although the SF + O group exhibited the lowest VEGF level among the treatment groups, no statistically significant difference was observed between the SF + O and ANT groups (p > 0.05).

A similar distribution was observed for TNF-α levels (p < 0.001). The highest TNF-α level was recorded in the PC group (4.980 ± 0.620 ng/mL), whereas the lowest level was observed in the NC group (2.910 ± 0.210 ng/mL). Compared with the PC group, TNF-α levels were significantly reduced in the SF (3.620 ± 0.480 ng/mL), O (3.710 ± 0.510 ng/mL), SF + O (3.340 ± 0.420 ng/mL), and ANT (3.520 ± 0.560 ng/mL) groups (p < 0.001). The SF + O group demonstrated the lowest mean TNF-α level among the treatment groups; however, no statistically significant difference was detected between the SF + O and ANT groups (p > 0.05).

Overall, inflammatory and angiogenic markers were highest in the PC group and lowest in the NC group. While the SF and O groups yielded comparable results, the SF + O combination was associated with a more pronounced reduction, particularly in VEGF and TNF-α levels (Figure 3).

### 3.3. Histopathological findings

**Figure 4:**
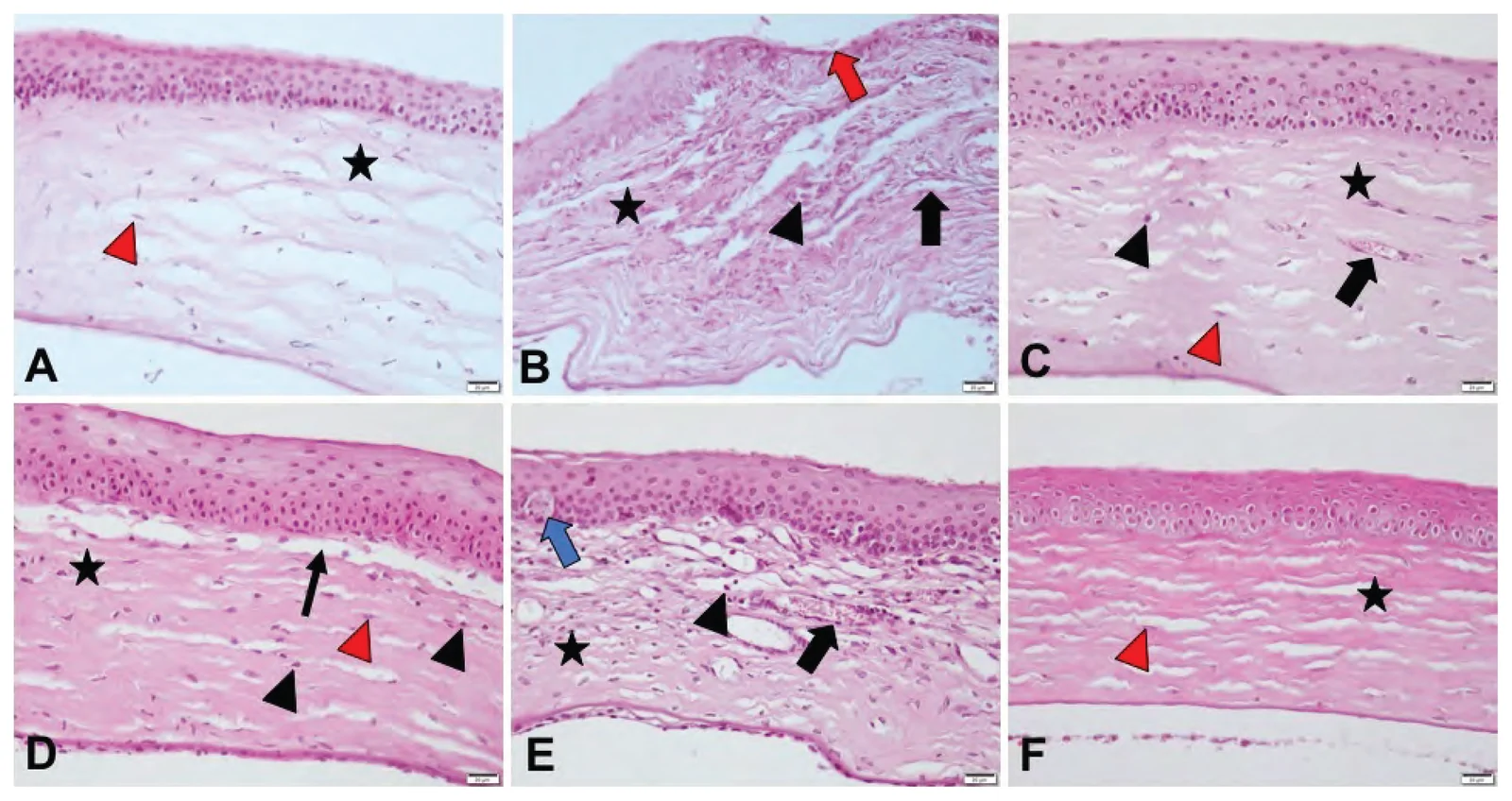
Light microscopic appearance of corneal morphology. (A) Negative control group; (B) Positive control group; (C) Silk fibroin + ozone group; (D) Silk fibroin group; (E) Ozone group; (F) Antibiotic group. Stroma (asterisk), fibroblasts (red arrowheads), inflammatory cells (black arrowheads), blood vessels (thick arrows), stromal separation (thin arrows), goblet cells (blue arrows), and epithelial loss (red arrows). Hematoxylin and eosin (H & E) staining, ×400 magnification. Scale bar: 20 μm.

Representative histopathological images are presented in Figure 4.

In the NC group, the corneal epithelium and stroma exhibited normal morphology. Collagen fibers were regularly arranged, and fibroblast morphology was preserved (Figure 4A).

In the PC group, marked epithelial loss and severe stromal damage were observed. The stroma showed pronounced edema, disorganization of collagen fibers, prominent neovascularization, and dense inflammatory cell infiltration (Figure 4B).

In the SF + O group, epithelial integrity was largely preserved, and mild epithelial thickening was evident. Mild stromal edema was present; however, the overall organization of collagen fibers was maintained. Limited inflammatory cell infiltration and focal neovascularization were observed. Stromal architecture appeared more organized compared with the other treatment groups (Figure 4C).

In the SF group, epithelial thickening and preservation of epithelial continuity were noted. The stroma exhibited mild-to-moderate edema and partial disorganization of collagen fibers. Focal stromal separations, limited neovascularization, and mild-to-moderate inflammatory cell infiltration were present (Figure 4D).

In the O group, epithelial thickening was observed, and epithelial integrity was largely maintained. Occasional goblet cell–like, mucin-containing cells were identified within the epithelial layer, suggesting epithelial differentiation changes. Mild-to-moderate stromal edema and partial collagen disorganization were detected. Mild inflammatory cell infiltration and focal neovascularization were present (Figure 4E).

In the ANT group, the corneal epithelium appeared close to normal morphology. Minimal stromal edema was observed, and neither inflammatory cell infiltration nor neovascularization was detected (Figure 4F).

### 3.4. Correlation between biochemical and histopathological findings

Histopathological findings were consistent with the biochemical results. The combination therapy (SF + O) was associated with more pronounced improvement compared with the single-treatment groups, corresponding to its stronger reduction in inflammatory and angiogenic markers. The SF and O groups exhibited comparable degrees of improvement, consistent with their similar biochemical profiles. In contrast, the PC group showed marked inflammation and tissue damage both biochemically and histologically.

## 4. Discussion

The present study demonstrates that hydrolyzed silk fibroin derived from *Bombyx hatayensis* and a commercially available ozonized gel, administered individually and in combination, significantly attenuate inflammatory and angiogenic responses following alkali-induced corneal injury in a rat model. To our knowledge, this is the first experimental investigation evaluating the ocular surface effects of hydrolyzed silk fibroin obtained from an endemic silkworm species in the setting of corneal alkali burns. Biochemically, all treatment groups showed significant reductions in TGF-β, VEGF, and TNF-α levels compared with untreated injured controls, while the combined SF + O therapy achieved the lowest VEGF and TNF-α levels among the treatment groups, with no statistically significant difference compared with the antibiotic-treated group. Histopathological findings were consistent with these molecular changes, as the SF + O group exhibited better preservation of epithelial integrity, reduced inflammatory cell infiltration, and improved stromal organization relative to the single-treatment groups. Collectively, these findings suggest that the integration of a naturally derived regenerative biomaterial with an oxidative stress–modulating agent may offer a biologically coherent and potentially additive or enhanced therapeutic effect for mitigating inflammation and neovascularization in severe chemical corneal injury. However, it should be noted that the present study did not directly investigate upstream signaling pathways such as NF-κB, HIF-1α, or oxidative stress–related mechanisms. Therefore, the observed effects should be interpreted as indirect modulation of the inflammatory and angiogenic microenvironment rather than direct pathway-specific inhibition.

The ocular safety of silk fibroin–based biomaterials has been extensively discussed in the literature, particularly with regard to immunogenicity, inflammatory responses, and theoretical amyloidogenic risks. Madden et al. [11] highlighted that, while purified silk fibroin demonstrates low cytotoxicity and favorable biocompatibility, concerns may arise in the context of long-term implantation or persistent inflammatory environments, particularly with solid or scaffold-based constructs. Importantly, these considerations primarily apply to implantable fibroin materials with prolonged tissue residence. In contrast, the present study utilized a topically applied hydrolyzed silk fibroin formulation characterized by low molecular weight and transient ocular surface exposure within an acute injury model. This formulation differs substantially in its physicochemical behavior and tissue interaction profile compared with permanent implants. Consistent with this distinction, no evidence of exaggerated inflammation, increased cytokine expression, or adverse histopathological responses was observed in the SF-treated groups. In contrast, hydrolyzed silk fibroin, alone and particularly in combination with ozone, was associated with significant reductions in TGF-β, VEGF, and TNF-α levels, accompanied by improved epithelial integrity and stromal organization. These findings support the short-term ocular safety profile of hydrolyzed silk fibroin in acute corneal injury and underscore the importance of formulation, route of administration, and exposure duration when evaluating biomaterial safety.

From a mechanistic perspective, corneal alkali burn–induced fibrosis is closely associated with sustained activation of TGF-β signaling, which promotes myofibroblast differentiation and extracellular matrix deposition. In the present study, significantly elevated TGF-β levels in the positive control (PC) group confirmed activation of this profibrotic pathway, whereas all treatment groups demonstrated marked suppression, particularly in the SF + O group.

These findings are consistent with previous studies highlighting the central role of TGF-β–mediated pathways in corneal fibrosis. For example, Donohoe et al. [12] showed that modulation of TGF-β–related signaling reduces inflammation and fibrosis through effects on macrophage activity, whereas Wang et al. [13] demonstrated attenuation of stromal injury via suppression of TGF-β–associated responses. In addition, AMPK-mediated inhibition of TGF-β1 has been shown to reduce myofibroblast differentiation and fibrotic remodeling [14].

In line with these reports, the reduced TGF-β levels observed in our study were accompanied by improved stromal organization and decreased inflammatory cell infiltration, particularly in the SF + O group. Importantly, these findings suggest that the therapeutic effect is associated with modulation of fibrosis-related pathways rather than direct pathway-specific inhibition.

The combined SF + O treatment produced the lowest VEGF and TNF-α levels among the treatment groups, suggesting concurrent suppression of both angiogenic and inflammatory mediators. Given the well-established interplay between TNF-α–driven inflammation and VEGF-mediated neovascularization, the dual reduction of these markers may have contributed to the improved stromal architecture and limited neovascularization observed histologically. The parallel improvement in molecular and structural parameters supports a biologically coherent therapeutic effect rather than an isolated biochemical alteration.

In contrast to pharmacological or cell-based strategies that directly target specific molecular pathways, the present study explores a comparatively simple and translational approach using topically applied cell-free hydrolyzed silk fibroin to modulate the corneal epithelial healing microenvironment. Although no direct inhibitors of TGF-β signaling were employed, hydrolyzed silk fibroin, particularly when combined with ozonized gel, was associated with significant reductions in TGF-β, VEGF, and TNF-α levels following alkali injury. These molecular changes were accompanied by improved epithelial integrity and more organized stromal architecture on histopathological examination. Rather than acting as a targeted molecular antagonist, hydrolyzed silk fibroin may contribute to the stabilization of the ocular surface environment, thereby indirectly attenuating inflammation-driven activation of profibrotic pathways. The enhanced biochemical suppression observed in the SF + O group further suggests that combining a regenerative biomaterial with an oxidative stress–modulating agent may improve the regulation of the inflammation–angiogenesis–fibrosis axis after chemical injury. Collectively, these findings support the concept that biomaterial-based modulation of the corneal healing milieu, even without direct pathway blockade, may influence key mediators involved in postalkali burn remodeling.

Pathological neovascularization following alkali injury is largely driven by excessive upregulation of VEGF, which represents a critical downstream mediator integrating inflammatory and stress-related signals within the injured cornea. Experimental evidence supports VEGF as a central effector in postburn angiogenesis. Wang et al. [15] demonstrated that targeting the ER stress/ROS–VEGF signaling axis using AdipoRon significantly reduced corneal neovascularization in an alkali burn model, underscoring the importance of VEGF-driven endothelial activation in chemical corneal injury. In the present study, VEGF levels were significantly elevated in untreated alkali-burned corneas, whereas all treatment groups exhibited marked reductions compared with the PC group. Notably, the combined SF + O therapy achieved the lowest mean VEGF level among the treatment groups, with values comparable to those observed in the ANT group. These molecular findings were accompanied histologically by reduced neovascularization and improved stromal organization, particularly in the SF + O group. Although the present approach does not involve direct pharmacologic VEGF inhibition, the observed attenuation of VEGF expression suggests that modulation of the inflammatory microenvironment may be sufficient to limit secondary angiogenic activation following alkali injury. Rather than acting as a classic anti-VEGF agent, the combination of hydrolyzed silk fibroin and ozone may influence the upstream inflammatory context in which VEGF is induced. This indirect regulatory profile may represent a biologically coherent strategy for reducing pathological angiogenesis while preserving physiological wound-healing processes.

Hypoxia-related signaling mechanisms are also known to contribute to angiogenesis after alkali injury, with hypoxia-inducible factor-1α (HIF-1α) acting as an upstream regulator of VEGF expression in ischemic and inflamed tissues. Experimental studies have explored strategies aimed at suppressing the HIF-1α–VEGF axis to limit postburn neovascularization [16]. These findings underscore the recognized role of hypoxia-driven pathways in corneal angiogenesis. While such approaches highlight the biological importance of VEGF-centered signaling, they often depend on gene interference techniques or invasive delivery systems. In the present study, although hypoxia-related mediators were not directly evaluated, significant reductions in VEGF levels were observed across all treatment groups compared with untreated injured controls, with the lowest mean VEGF levels observed in the SF + O group. These biochemical findings were accompanied by reduced neovascularization on histopathological examination. Rather than directly targeting specific transcriptional regulators such as HIF-1α, the combination of hydrolyzed silk fibroin and ozonized gel may modulate the inflammatory and stress-associated microenvironment in which VEGF expression is induced. This indirect attenuation of VEGF-driven angiogenic signaling suggests that stabilization of the corneal surface milieu may be sufficient to mitigate secondary neovascular responses following alkali injury.

TNF-α is a pivotal mediator in corneal alkali injury, sustaining inflammatory signaling, oxidative stress, and secondary epithelial damage. Experimental studies have consistently demonstrated marked upregulation of TNF-α following alkali burns, highlighting its central role in persistent tissue injury [17]. Beyond its general inflammatory function, TNF-α has been implicated in limbal stem cell damage through monocyte recruitment and apoptosis, thereby contributing to impaired reepithelialization and long-term ocular surface dysfunction [18]. Moreover, early TNF-α inhibition has been shown to reduce neovascularization, inflammatory cell infiltration, and tissue apoptosis in experimental alkali burn models [19], underscoring the therapeutic relevance of modulating this cytokine. In the present study, TNF-α levels were significantly elevated in the untreated alkali-burn group, confirming activation of this inflammatory axis. All treatment groups demonstrated significant reductions in TNF-α compared with PC group, with the lowest mean levels observed in the SF + O group. These biochemical findings were consistent with histopathological observations showing reduced inflammatory cell infiltration and improved stromal organization, particularly in the SF + O group. While the present approach does not involve direct TNF-α neutralization, the attenuation of TNF-α expression suggests effective modulation of inflammation-associated signaling cascades within the injured corneal microenvironment. The comparable TNF-α levels between the SF + O and ANT groups further suggest that the combination therapy achieved antiinflammatory efficacy similar to conventional topical management in this acute injury setting.

Evidence from nonocular tissue engineering applications further supports the broader concept that silk fibroin–based platforms can interact with growth factor–related pathways involved in angiogenesis and fibrosis. In a vascular tissue engineering model, Liu et al. [20] demonstrated that a silk fibroin–based construct incorporating sequential delivery of VEGF and TGF-β1 inhibitors promoted tissue integration while limiting excessive collagen deposition and pathological remodeling. Although that study employed engineered inhibitor-loaded constructs and was conducted outside the ocular surface, it highlights the capacity of silk fibroin platforms to influence biological processes associated with vascularization and fibrotic repair. In contrast to engineered delivery systems, the present study utilized a cell-free topically applied hydrolyzed silk fibroin formulation without incorporated molecular inhibitors. Despite this simpler approach, significant reductions in TGF-β and VEGF levels were observed following treatment, particularly in the SF + O group, and were accompanied by improved stromal organization and reduced neovascularization. These findings suggest that hydrolyzed silk fibroin may contribute to the modulation of the postinjury corneal microenvironment through indirect regulation of inflammatory and profibrotic mediators. Rather than functioning solely as a passive structural scaffold, silk fibroin in this context may participate in shaping the biological milieu of corneal healing, especially when combined with an oxidative stress–modulating agent such as ozone.

The present study has several noteworthy strengths. First, it provides the first experimental evaluation of hydrolyzed silk fibroin derived from the endemic silkworm species *Bombyx hatayensis* in a corneal alkali burn model, introducing a region-specific, naturally sourced biomaterial into ocular surface research. Second, the study integrates clinical evaluation, quantitative biochemical analysis (TGF-β, VEGF, TNF-α), and histopathological assessment, enabling a multidimensional interpretation of therapeutic effects. The concordance between molecular suppression and structural improvement strengthens the internal consistency of the findings. Third, the inclusion of both single-treatment groups and a combination therapy arm allowed comparative assessment of potential additive effects, revealing that SF + O achieved the lowest VEGF and TNF-α levels among the treatment groups and outcomes comparable to those of conventional topical antibiotic therapy. Finally, the use of a cell-free topically administered formulation enhances translational relevance, as the approach avoids invasive delivery systems, gene-based modulation, or exogenous growth factor blockade. Despite these strengths, several limitations should be acknowledged. No formal a priori power calculation was performed, as the study was designed as an exploratory preclinical investigation. In addition, the relatively small sample size, particularly in the negative control group, may limit statistical power and generalizability. However, the negative control group was included primarily as a baseline reference without injury and was therefore designed with a reduced sample size in accordance with ethical reduction principles. The follow-up period was limited to 21 days and did not evaluate long-term fibrosis or recurrence of neovascularization. Only selected inflammatory and angiogenic mediators were measured, and upstream molecular signaling pathways were not directly investigated. In addition, histopathological evaluation was performed descriptively without a standardized quantitative or semiquantitative scoring system, which may limit the objectivity of morphological comparisons. Finally, a single concentration and dosing regimen were tested, precluding dose–response analysis. Further translational and long-term safety studies are required before clinical application can be considered.

## 5. Conclusion

In conclusion, topically applied hydrolyzed silk fibroin derived from the endemic silkworm species *Bombyx hatayensis*, administered alone or in combination with ozonized gel, significantly attenuated inflammatory and angiogenic responses in a rat model of corneal alkali burn. Treatment was associated with the suppression of key mediators, including TGF-β, VEGF, and TNF-α, alongside improved epithelial integrity and more organized stromal architecture. Notably, the combined SF + O therapy demonstrated the most favorable biochemical and histopathological profile among the treatment groups, achieving outcomes similar to those of conventional topical management, without statistically significant differences. These findings suggest that hydrolyzed silk fibroin–based formulations may represent a promising, biocompatible, and cell-free adjunctive strategy for modulating the postinjury corneal microenvironment in severe chemical burns. Further long-term and translational studies are warranted to confirm these findings.

## Figures and Tables

**Table:** Corneal opacity scores (Yoeruek 0–4 Scale) and neovascularization scores.

Group	Day 1	Day 4	Day 7	Day 21
**NC (n = 3)**	0.0 ± 0.0 / 0.0 ± 0.0	0.0 ± 0.0 / 0.0 ± 0.0	0.0 ± 0.0 / 0.0 ± 0.0	0.0 ± 0.0 / 0.0 ± 0.0
**PC (n = 7)**	3.6 ± 0.5 / 1.2 ± 0.4	3.4 ± 0.5 / 2.3 ± 0.5	3.3 ± 0.5 / 3.0 ± 0.5	3.2 ± 0.6 / 3.4 ± 0.6
**SF (n = 7)**	3.5 ± 0.5 / 1.1 ± 0.3	2.8 ± 0.6 / 1.8 ± 0.4	2.4 ± 0.5 / 2.2 ± 0.5	2.1 ± 0.6 / 2.5 ± 0.5
**O (n = 7)**	3.4 ± 0.5 / 1.1 ± 0.3	2.9 ± 0.5 / 1.9 ± 0.4	2.3 ± 0.6 / 2.1 ± 0.4	2.0 ± 0.5 / 2.4 ± 0.5
**SF + O (n = 7)**	3.5 ± 0.5 / 1.0 ± 0.3	2.4 ± 0.5 / 1.5 ± 0.4	1.9 ± 0.4 / 1.7 ± 0.4	1.4 ± 0.5 / 1.8 ± 0.4
**ANT (n = 7)**	3.4 ± 0.6 / 1.0 ± 0.3	2.3 ± 0.6 / 1.4 ± 0.4	1.8 ± 0.5 / 1.6 ± 0.4	1.3 ± 0.4 / 1.7 ± 0.3
**p value**	0.74 / 0.81	<0.001 / <0.001	<0.001 / <0.001	<0.001 / <0.001

Data are presented as mean ± standard deviation. Corneal opacity was graded using the Yoeruek 0–4 scale. Corneal neovascularization was graded on a 0–4 scale according to the centripetal extent of newly formed vessels extending from the limbus toward the central cornea. Values are presented as opacity score/neovascularization score. Significant intergroup differences were observed from day 4 onward (p < 0.001). The SF + O and ANT groups demonstrated significantly lower opacity and neovascularization scores compared with the PC group on days 7 and 21 (p < 0.001), with no statistically significant difference between the SF + O and ANT groups (p > 0.05).

## Data Availability

The datasets generated and/or analyzed during the current study are available from the corresponding author upon reasonable request.

## References

[1] Chang JH Gabison EE Kato T Azar DT Corneal neovascularization Current Opinion in Ophthalmology 2001 12 4 242249 10.1097/00055735-200108000-00002 11507336

[2] Zhang X Shen L Jin Y Saban DR Chauhan SK Depletion of passenger leukocytes from corneal grafts: an effective means of promoting transplant survival? Investigative Ophthalmolology and Visual Science 2009 50 7 3137 3144 10.1167/iovs.08-1899 PMC2739689 19136708

[3] Lu P Li L Liu G Zhang X Mukaida N Enhanced experimental corneal neovascularization along with aberrant angiogenic factor expression in the absence of IL-1 receptor antagonist Investigative Ophthalmology and Visual Science 2009 50 10 4761 4768 10.1167/iovs.08-2732 19458323

[4] Amano S Rohan R Kuroki M Tolentino M Adamis AP Requirement for vascular endothelial growth factor in wound- and inflammation-related corneal neovascularization Investigative Ophthalmology and Visual Science 1998 39 1 18 22 9430540

[5] Kim DR Park SK Kim EJ Kim DK Yoon YC Dexamethasone acetate loaded poly(ɛ-caprolactone) nanofibers for rat corneal chemical burn treatment Scientific Reports 2024 14 21806 10.1038/s41598-024-62026-x 39300144 PMC11413004

[6] Ikebukuro T Arima T Kasamatsu M Nakano Y Tobita Y Disulfiram Ophthalmic Solution Inhibited Macrophage Infiltration by Suppressing Macrophage Pseudopodia Formation in a Rat Corneal Alkali Burn Model International Journal of Molecular Sciences 2023 24 1 735 10.3390/ijms24010735 36614177 PMC9821574

[7] Lyu Y Liu Y He H Wang H Application of Silk-Fibroin-Based Hydrogels in Tissue Engineering Gels 2023 9 5 431 10.3390/gels9050431 37233022 PMC10218181

[8] Aydoğan S Erol H Baran M Effects of ozone therapy on acidic corneal burns in rats Veterinary research forum: an international quarterly journal 14 4 195 199 10.30466/vrf.2022.551199.3432 PMC10170467 37181857

[9] Ammassam Veettil R Li W Pflugfelder SC Koch DD A Mouse Model for Corneal Neovascularization by Alkali Burn Journal of Visualized Experiments: JoVE 2023 Jun 30 196 10.3791/65289 37458425

[10] Yoeruek E Ziemssen F Henke-Fahle S Tatar O Tura A Safety, penetration and efficacy of topically applied bevacizumab: evaluation of eyedrops in corneal neovascularization after chemical burn Acta Ophthalmologica 2008 86 322 328 10.1111/j.1755-3768.2007.01049.x 17995975

[11] Madden PW Klyubin I Ahearne MJ Silk fibroin safety in the eye: a review that highlights a concern BMJ Open Ophthalmology 2020 Sep 23 5 1 e000510 10.1136/bmjophth-2020-000510 PMC7513638 33024827

[12] Donohoe E Canning A Johnston E Moosavizadeh S Wang J Small extracellular vesicles secreted from TGF-β1-licensed mesenchymal stromal cells reduce inflammation-associated injury following corneal alkali burn Stem cell Research and Therapy 16 1 376 10.1186/s13287-025-04661-3 PMC12261806 40660344

[13] Wang H Guo Z Liu P Yang X Li Y Luteolin ameliorates cornea stromal collagen degradation and inflammatory damage in rats with corneal alkali burn Experimental Eye Research 231 109466 10.1016/j.exer.2023.109466 37059215

[14] Nuwormegbe SA Kim SW AMPK Activation by 5-Amino-4-Imidazole Carboxamide Riboside-1-β-D-Ribofuranoside Attenuates Alkali Injury- Induced Corneal Fibrosis Investigative Ophthalmology and Visual Science 61 6 43 10.1167/iovs.61.6.43 PMC7415321 32561924

[15] Wang C Yuan K Wu Y Jiang T Mou Y Targeting ER stress/ROS-driven VEGF signaling axis: AdipoRon as a multifunctional therapeutic agent for alkali burn-induced corneal neovascularization Free Radical Biology and Medicine 241 117 136 10.1016/j.freeradbiomed.2025.09.012 40939851

[16] Fu YC Xin ZM Inhibited corneal neovascularization in rabbits following corneal alkali burn by double-target interference for VEGF and HIF-1α Bioscience Reports 39 1 BSR20180552 10.1042/BSR-2018-0552_RET PMC6356011 30355648

[17] Li Y Yang YQ Lin Y Yan K Lyu YF Alkali burn injury model of meibomian gland dysfunction in mice International journal of Ophthalmology 17 12 2158 2166 10.18240/ijo.2024.12.02 PMC11589446 39697880

[18] Zhou C Lei F Mittermaier M Dana R Dohlman CH TNF-α Suppression Attenuates Limbal Stem Cell Damage in Ocular Injury Cornea 44 6 762 771 10.1097/ICO.0000000000003738 PMC12052064 39626088

[19] Cade F Paschalis EI Regatieri CV Vavvas DG Dana R Alkali burn to the eye: protection using TNF-α inhibition Cornea 33 4 382 389 10.1097/ICO.0000000000000071 24488127

[20] Liu Z Rütten S Buhl EM Zhang M Liu J Development of a Silk Fibroin-Small Intestinal Submucosa Small-Diameter Vascular Graft with Sequential VEGF and TGF-β1 Inhibitor Delivery for In Situ Tissue Engineering Macromolecular Bioscience 23 9 e2300184 10.1002/mabi.202300184 37262314

